# Targeting the Glucose–Insulin Link in Head and Neck Squamous Cell Carcinoma Induces Cytotoxic Oxidative Stress and Inhibits Cancer Growth

**DOI:** 10.1158/2767-9764.CRC-23-0506

**Published:** 2025-06-06

**Authors:** Simbarashe Mazambani, Seong-Ho Park, Joshua H. Choe, An H. Nguyen, Bok-Soon Lee, Ji Yun Jeong, Shin Yup Lee, Chul-Ho Kim, Yea-In Park, Joselyn Padilla, Jiyoung Lee, Dinesh Thotala, Tae Gyu Oh, Pankaj K. Singh, Hoon Hur, Junho K. Hur, Jung-whan Kim, Tae Hoon Kim

**Affiliations:** 1 Department of Biological Sciences, The University of Texas at Dallas, Richardson, Texas.; 2 Department of Oncology Science, The University of Oklahoma College of Medicine, Stephenson Cancer Center, Oklahoma City, Oklahoma.; 3 Department of Medicine, Graduate School, Hanyang University, Seoul, Korea.; 4 Division of Medical Sciences, Harvard Medical School, Boston, Massachusetts.; 5 Department of Medical Oncology, Dana-Farber Cancer Institute, Boston, Massachusetts.; 6 Department of Otolaryngology, Ajou University School of Medicine, Suwon, Korea.; 7 Department of Pathology, Kyungpook National University School of Medicine, Daegu, Korea.; 8 Department of Internal Medicine, Kyungpook National University School of Medicine, Daegu, Korea.; 9 Department of Biochemistry & Molecular Medicine, George Washington University School of Medicine and Health Sciences, GW Cancer Center, Washington, District of Columbia.; 10 Department of Radiation Oncology, The University of Oklahoma College of Medicine, Stephenson Cancer Center, Oklahoma City, Oklahoma.; 11 Department of Surgery, Ajou University School of Medicine and Cancer Biology Graduate Program, Ajou University Graduate School of Medicine, Suwon, Korea.; 12 Department of Genetics, College of Medicine, Hanyang University, Seoul, Korea.; 13 Graduate School of Biomedical Science and Engineering, Hanyang University, Seoul, Korea.; 14 Department of Comparative Medicine, Medical University of South Carolina, Charleston, South Carolina.

## Abstract

Head and neck squamous cell carcinoma (HNSCC) remains a clinically challenging malignancy with limited targeted therapy options and poor patient outcomes. Thus, identifying unique dependencies, including HNSCC-specific metabolic reprogramming, is imperative for improving treatment strategies for this disease. In this study, we show that HNSCC relies on elevated glucose transporter 1 (GLUT1)–mediated glucose uptake to support redox homeostasis and tumor growth. Analyses of GLUT1 expression data in tumors and cancer cell lines reveal significant upregulation of GLUT1 in HNSCC relative to both normal tissue and other cancer subtypes and that high GLUT1 expression correlates with poorer clinical outcomes. Using a basal epithelial layer–specific GLUT1-knockout mouse model, we demonstrate that GLUT1 ablation in HNSCC cells of origin markedly attenuates tumor initiation and progression, implicating the necessity of GLUT1 in HNSCC tumorigenesis. Building on this observation, combining pharmacologic inhibition of GLUT1 with pro-oxidants such as vitamin C and auranofin induces potent cytotoxicity *in vitro* and *in vivo*, partly by precipitating oxidative stress. We further observe that insulin signaling is required to sustain glucose uptake and redox homeostasis, as insulin receptor knockdown decreases proliferation and increases reactive oxygen species levels. Together, these results suggest that although GLUT1 overexpression is a key driver of glucose uptake, insulin signaling also contributes to the metabolic and oncogenic pathways underlying HNSCC progression. Consequently, strategies that co-target GLUT1 and insulin signaling to restrict glucose flux may synergize with pro-oxidant therapies, offering a promising therapeutic avenue for HNSCC.

**Significance:**

Enhanced GLUT1 expression and oncogenic insulin signaling drive elevated glucose uptake in HNSCC, which contribute to the maintenance of redox homeostasis and tumor growth. Disrupting both glucose uptake and redox balance may offer a promising therapeutic approach.

## Introduction

Head and neck squamous cell carcinoma (HNSCC) is the sixth most common cancer worldwide and a leading cause of cancer mortality, with a reported 890,000 new cases and 450,000 deaths in 2018 (1). HNSCCs arise from the mucosal epithelium of the oral cavity, pharyngeal regions (nasopharynx, oropharynx, and hypopharynx), and larynx (2). Epidemiologic studies report a diverse range of risk factors for HNSCC, including tobacco usage, alcohol consumption, exposure to environmental pollutants, and infection with viral agents such as human papillomavirus (HPV) and Epstein-Barr Virus (1, 2). HNSCC is often diagnosed at late stages because of the lack of effective screening strategies and exhibits marked heterogeneity, thereby contributing to difficulty in treatment (3, 4). Thus, aggressive multimodal treatment approaches are often necessary and have traditionally involved surgery accompanied by adjuvant radiotherapy or chemotherapy (1, 5). Recent advances in systemic therapies, especially with the approval of PD-1/PD-L1 antagonists with or without chemotherapy in the first-line treatment for unresectable disease or metastatic HNSCC, have yielded better responses (1, 5–10). Despite these advances in the therapeutic landscape, not all patients may benefit from immune checkpoint inhibitors and the survival of patients with HNSCC has remained largely unchanged over the last several decades (1, 4). Therefore, the development of targeted strategies that are more effective and tolerable, especially for patients who might not benefit from current regimens, remains a pressing need that is currently being explored (3, 11, 12).

Understanding the unique metabolic signatures of HNSCCs can pave the way for such targeted interventions. Although the HNSCC landscape has been shaped by conventional and immune-based therapeutic approaches, exploring the cancer’s metabolic vulnerabilities offers a comprehensive and complementary avenue. Our recent work showed an exquisite dependence of squamous cell carcinomas (SCC) on glucose, driven by highly upregulated glucose transporter 1 (GLUT1) expression (13). We demonstrated that GLUT1-mediated glucose reliance is a defining phenotype and a unifying feature of all SCCs and established that enhanced glycolytic metabolism is an integral process to fuel the anabolic demands of enhanced growth, maintain redox homeostasis, and promote the continued survival of SCCs. Increased glucose influx into squamous cancer cells enhances their antioxidative capacity via the pentose phosphate pathway (PPP), which generates reducing equivalents in the form of NADPH, and *de novo* serine biosynthesis, which supports glutathione synthesis by supplying glycine (14). Given that mutagenic, but not cytotoxic, levels of oxidative stress are often key initiators of cancerous lesions, especially for SCCs, and as tumors frequently experience high reactive oxygen species (ROS) because of both elevated metabolic activity and hypoxia, SCCs may adapt by enhancing antioxidant systems (10, 15). This adaptation may render tumors more sensitive to stimuli that disrupt the redox balance necessary to maintain tumorigenic potential. Although various studies have debated the efficacy of pro-oxidants as cancer therapies (16–20), data on the overall benefit of these therapies in patients with HNSCC remain largely inconclusive. We sought to investigate whether HNSCCs might be susceptible to elevated cytotoxic ROS via combinatorial inhibition of glucose influx and treatment with therapeutic pro-oxidants (21).

Although the cell-intrinsic modulation of redox balance through glucose metabolism provides a potential therapeutic avenue, examining the contribution of upstream regulators that influence glucose uptake and use remains critical. Insulin signaling is one such regulator that has emerged as a major player in tumorigenesis. In fact, hyperinsulinemia has been linked to early tumor development as well as tumor progression and poorer survival in certain patients with cancer (22–26). Upon insulin binding, insulin-like growth factor receptors activate the PI3K and MAPK pathways, which regulate cell proliferation, survival, migration, metabolism, and angiogenesis (27–29). This becomes particularly pertinent in HNSCC, in which an amplification of *PIK3CA*, encoding the PI3K catalytic component, is observed in about 30% of cases. PI3K–AKT pathway activation not only regulates glucose uptake by driving GLUT1 plasma membrane translocation but also transcriptionally and posttranslationally controls glucose use (22–24, 30). Our prior investigations revealed that dampening insulin signaling by restricting glucose uptake stymied SCC tumor growth, chiefly by disrupting glucose metabolism and suppressing the PI3K/AKT axis (9, 25). Such findings compelled us to explore further this glucose–insulin nexus, which may be especially relevant in the context of patients with diabetic SCC. Our present study highlights how the confluence of oncogenic insulin signaling and glucose metabolism sustains the antioxidant requirements pivotal for squamous cancer cell growth and survival.

## Materials and Methods

### Mice

C57BL/6 (RRID: IMSR_JAX:000664) and NOD/SCID (RRID: IMSR_JAX:001303) mice were obtained from The Jackson Laboratory. All experimental procedures using mice were approved by The University of Texas at Dallas and the University of Oklahoma Health Sciences Center Institutional Animal Care and Use Committee. Both male and female mice were included in all experiments. For both subcutaneous xenograft and 4-nitroquinoline 1-oxide (4NQO)–induced HNSCC models, tumor-bearing mice were randomly assigned into experimental groups.

### 4NQO-induced head and neck and esophageal cancer models

4NQO was initially dissolved in propylene glycol at 10 mg/mL and diluted in drinking water to a final concentration of 100 μg/mL for administration (31). Mice were treated with 4NQO for a maximum treatment time of 8 weeks and then observed for up to an additional 20 weeks after treatment. Briefly, after 8 weeks, 4NQO was removed from the drinking water and maintained with normal drinking water for an additional 4 weeks (a total 12 weeks after initial exposure to 4NQO). Mice were then injected intraperitoneally with tamoxifen (100 mg/kg once daily for 5 consecutive days) to delete the GLUT1 gene in the Krt5^+^ basal progenitor cells. dTomato, included as a fluorescent reporter of Cre activity in our model system, was activated specifically in GLUT1-expressing basal epithelial cells upon treatment of mice with tamoxifen. Control mice were given normal water with a vehicle of propylene glycol for the entire treatment period. All mice were given water + 4NQO or water + vehicle *ad libitum*. During the treatment period, animals were monitored daily and weighed weekly. The oral mucosa of each mouse was examined under inhalation anesthesia weekly to check for oral tumors. Food intake was closely monitored for animals developing esophageal tumors. Mice were euthanized immediately when they showed signs of stress, weight loss, or discomfort. To provide a comprehensive temporal analysis of cancer progression and severity, mice were euthanized and their tongues and esophagi were collected for analysis.

### Basal epithelial cell–specific GLUT1 knockout

Keratin 5 (Krt5) is an intermediate filament protein specifically expressed in the basal layer of stratified epithelial cells from which premalignant dysplastic squamous lesions arise. To generate a conditional deletion of GLUT1 in the basal squamous epithelium, Krt5:Cre mice were crossed with GLUT1 floxed (*Glut1*^*loxP/loxP*^) mice. We also introduced a heterozygous deletion of the tumor suppressor p53, which mimics the loss of heterozygosity that is common in HNSCC. In our model, *Krt5:CreER*; *LSL-dTomato*; *Trp53*^*+/−*^; *Glut1*^*loxP/loxP*^ mice express tamoxifen-inducible Cre recombinase under the direction of the *Krt5* promoter. Cre activity was assessed using the fluorescent tomato reporter dTomato. After the administration of tamoxifen (100 mg/kg once daily for 5 consecutive days), the gene encoding GLUT1 was knocked out in the basal, Krt5+ squamous epithelium.

### Cell lines

The SCC-61 HNSCC cell line (RRID: CVCL_7118) was provided by Jeffrey Myers (MD Anderson Cancer Center). The FaDu HNSCC cell line was purchased from ATCC (RRID: CVCL_1218). Cells were cultured in high-glucose DMEM (Sigma-Aldrich) supplemented with 10% FBS (Sigma-Aldrich), 1% penicillin/streptomycin (Sigma-Aldrich), and 1% nonessential amino acids (Sigma-Aldrich; unless otherwise stated) at 37°C in a humidified 5% CO_2_ environment. All cell lines were tested for *Mycoplasma* monthly using the e-Myco kit (Boca Scientific). All cell lines were cultured for no more than 15 passages.

### Irradiation

Cell cultures were irradiated using the X-Rad 320 Biological Irradiator (320 kVp, 12.5 mA, and 2-mm Al filter) at a dose rate of ∼2 Gy/minute. Cells were positioned centrally in the irradiator chamber to ensure uniform exposure. Radiation doses were delivered according to experimental requirements (4 Gy) with exposure times adjusted accordingly. After irradiation, cells were immediately returned to the incubator.

### Colony formation assay

The following cell lines were seeded at indicated densities (FaDu: 3,000 cells/well; Detroit 562: 1,000 cells/well) in six-well plates in complete medium. Cells were treated with GLUT1 inhibitors (20 μmol/L WZB117 and 100 nmol/L BAY-876) 24 hours after seeding with or without 4 Gy of X-ray radiation. The media was replaced 72 hours after treatment with GLUT1 inhibitor-free complete medium. Subsequently, media was changed every 3 days. The cells were washed with PBS 10 days after treatment and fixed with 100% methanol for 15 minutes. They were stained using 0.5% crystal violet for 20 minutes, washed, and air dried. The colony area was quantified using ImageJ software.

### Pro-oxidant vitamin C experiments

To pharmacologically inhibit GLUT1, we used the well-characterized irreversible GLUT1 inhibitors WZB117 and BAY-876 to target GLUT1-mediated glucose influx and its related function in redox homeostasis (32–35). The specificity of these GLUT1 inhibitors has been previously well validated and demonstrated in our previous studies. We combined these GLUT1 inhibitors with pro-oxidant high-dose vitamin C to potently disable cellular oxidative defense machinery. Various cell lines were treated with WZB117 (10–50 μmol/L) or BAY-876 (10–100 nmol/L) and with vitamin C (0–1 mmol/L). After treatment, glucose influx was evaluated using glucose uptake assays. Cellular oxidative stress was examined by measuring intracellular H_2_O_2_ (H_2_DCFDA staining).

### *In vivo* tumor xenograft experiments

For this experiment, 5 × 10^6^ cells suspended in 50% Matrigel (Corning Life Sciences) and 50% Hank’s Balanced Salt Solution (HBSS, Sigma-Aldrich) was subcutaneously implanted into the flank of NOD/SCID mice (The Jackson Laboratory) between 4 and 6 weeks of age. WZB117 (Calbiochem) 10 mg/kg was administered intraperitoneally once daily. Tumor volume was measured at indicated times using electronic calipers and estimated using the modified ellipsoid formula tumor volume = (length × width^2^)/2.

### GLUT1 inhibition *in vivo*

Mice were divided into four groups and received (i) vehicle control, (ii) WZB117 (10–20 mg/kg i.p. daily) or BAY-876 (3–5 mg/kg p.o. daily) only, (iii) vitamin C (4 g/kg i.p. daily) only, and combination of WZB117/BAY-876 and vitamin C. Subsequently, to evaluate physiologic effects of our proposed treatments, xenografts were established in SCID mice.

### Streptozotocin diabetes mouse model

Six-week-old mice were individually marked and weighed, and their baseline blood glucose levels were determined using a *OneTouch Ultra* blood glucose monitor. Next, mice received intraperitoneal injections of 40 mg streptozotocin (STZ)/kilogram of body weight daily (for NOD/SCID gamma and NOD/SCID males) for five consecutive days; age-matched controls received buffer-only injections. STZ was dissolved in sodium citrate buffer. Food and water were available *ad libitum*. The mice were observed daily, and blood glucose measurements were taken 1 week after the final injection. Mice with blood glucose levels exceeding 250 mg/dL were considered diabetic. HNSCC cell line xenografts were then established in diabetic and control mice. Tumor measurements were made for up to 3 weeks, at which point tumors were excised for histopathologic, protein, and RNA analyses. Prior to the final tumor measurement, mice were evaluated to see if they were still diabetic. Plasma was also collected for Insulin ELISA analysis.

### Blood glucose and insulin measurement

Blood collected from the tail of mice that were fasted for 6 hours prior was used to measure blood glucose via glucometer (OneTouch Ultra2). Up to 200 μL of blood was collected from the tail into EDTA-coated microfuge tube and centrifuged to isolate plasma for insulin measurement. Insulin levels were determined via Mouse Insulin ELISA Assay Kit (Crystal Chem) according to the manufacturer’s instruction.

### Immunoblotting

Cells were lysed by RIPA lysis buffer supplemented with cOmplete Protease Inhibitor (Roche) and subsequently with 20% amplitude sonication for 5 seconds, and lysates were cleared by 14,000 rpm centrifugation at 4°C for 15 minutes. Equivalent lysates were separated by SDS-PAGE and transferred onto polyvinylidene difluoride membranes (Thermo Fisher Scientific). Membranes after blocking in 5% nonfat dry milk dissolved in Tris-buffered saline with Tween 20 for 30 minutes were incubated in primary antibody diluted in 5% BSA overnight. Horseradish peroxidase–conjugated secondary antibodies diluted 1:5,000 in 5% nonfat milk were used and visualized using SuperSignal West Pico or Femto substrate kits (Thermo Fisher Scientific). The following commercial primary antibodies supplemented with 0.02% sodium azide were used for immunoblot analysis: p63 (1:1,000; Biocare Medical, Cat. #CM 163 A, RRID: AB_10582730), GLUT1 (1:1,000; Alpha Diagnostic International, Cat. #GT11-A, RRID: AB_2895172), Tyr1361 phosphorylated insulin receptor (Tyr1361-pINSR; 1:1,000; Thermo Fisher Scientific, Cat #PA5-38283, RRID: AB_2554884), insulin receptor (INSR; 1:1,000; Thermo Fisher Scientific, Cat. #AHR0271, RRID: AB_2536351), Ser473-pAKT (1:1,000; Cell Signaling Technology, Cat. #4058), AKT (1:1,000; Cell Signaling Technology, Cat. #9272, RRID: AB_329827), Ser235/236-pS6 (1:1,000; Cell Signaling Technology, Cat. #4858, RRID: AB_916156), S6 Ribosomal Protein (1:1,000; Cell Signaling Technology, Cat. #2217, RRID: AB_331355), Thr37/46-p4EBP1 (1:1,000; Cell Signaling Technology, Cat. #2855, RRID: AB_560835), cleaved caspase-3 (CC3; 1:1,000; Cell Signaling Technology, Cat. #9664, RRID: AB_2070042), and β-actin (1:5,000; Sigma-Aldrich, Cat. #A5441, RRID: AB_476744).

### IHC and immunofluorescence

Xenograft mice were perfused with 10 mmol/L EDTA in PBS followed by 4% paraformaldehyde. Xenograft tumors were extracted and fixed in 4% paraformaldehyde for 12 hours and then embedded in paraffin. Tissue blocks were then sectioned (5 μm) and subjected to heat-mediated antigen retrieval (citrate buffer, pH 6.0). Goat serum (Sigma-Aldrich) or donkey serum (Sigma-Aldrich) was used to block for 1 hour, and diluted primary antibodies were applied at 4°C overnight. VECTASTAIN ABC (VectorLabs) with DAB substrate (VectorLabs) was used to optimize staining according to the manufacturer’s protocol. The following primary antibodies were used: p63 (1:200; Biocare Medical; Cat. #CM 163 A), p63 (1:100; R&D Systems, Cat. #AF-1916), GLUT1 (1:250; Alpha Diagnostic International, GT11-A), Ki67 (1:500; Cell Signaling Technology, Cat. #12202), CC3 (1:200; Cell Signaling Technology, Cat. #9664), Ser473-pAKT (1:500; Cell Signaling Technology, Cat. #4058), Ser235/236-pS6 (1:200; Cell Signaling Technology, Cat. #4858), Ser139-pHistone H2A.X (1:1,000; Cell Signaling Technology, Cat. #9718), 4-hydroxynonenal (4HNE; 1:500; Abcam, ab46545), and CK5 (1:200; Abcam, ab52635). Images were taken using a Nikon ECLIPSE Ni-U microscope with NIS-Elements imaging software (Nikon) and quantified using ImageJ software (RRID: SCR_003070).

### *In vitro* ROS measurement

ROS levels were detected using the ROS Detection Cell-Based Assay Kit (DCFDA) or ROS-Glo H_2_O_2_ Assay Kit (Promega). Following cell preparation and incubation on 96-well plates according to the manufacturer’s protocols, fluorescent intensity or luminescence was measured using a BioTek Synergy H4 plate reader. Raw fluorescence and luminescence were normalized to cell count measured using a TC20 Automated Cell Counter (Bio-Rad).

### *In vitro* metabolic analysis

Glucose uptake was measured using the Glucose Uptake Cell-Based Assay Kit (Cayman Chemical) or Glucose Uptake-Glo Assay Kit (Promega) according to the manufacturer’s protocols. Following cell preparation and incubation with a fluorescent glucose analogue, 2-(N-(7-nitrobenz-2-oxa-1,3-diazol-4-yl)amino)-2-deoxyglucose, or 2-deoxy-D-glucose in glucose-free DMEM (Gibco) at 37°C in 96-well plates, fluorescent intensity or luminescence was measured using a BioTek Synergy H4 plate reader. Raw fluorescence and luminescence were normalized to cell count measured by a TC20 Automated Cell Counter (Bio-Rad).

### Short hairpin RNA and vectors

The following pLKO.1 short hairpin RNAs (shRNA) were used: shINSR#1 (Mission TRC shRNA, TRCN0000010523, Sigma-Aldrich), shINSR#2 (Mission TRC shRNA, TRCN0000000379, Sigma-Aldrich), shGLUT1 (Mission TRC shRNA, TRCN0000043583, Sigma-Aldrich), shIGFR1#1 (Mission shRNA, TRCN0000039677, Sigma-Aldrich), and shIGFR1#2 (Mission TRC shRNA, TRCN0000000422, Sigma-Aldrich). For lentivirus production, HEK293T cells (RRID: CVCL_0063) were transfected with viral packaging plasmids psPAX2 (RRID: Addgene_12260) and pMD2.G (RRID: Addgene_12259), and pLKO.1 (RRID: Addgene_139470) shRNA using Lipofectamine 3000 (Invitrogen). Cells were incubated with viral supernatant containing polybrene (8 mg/mL). pLKO.1-shScr (RRID: Addgene_17920) was used as the control vector. Targeting sequences for all shRNAs are provided in Supplementary Table S1. pLenti CMV GFP Blast (659-1) was a gift from Eric Campeau and Paul Kaufman (Addgene, plasmid # 17445). pHAGE-PIK3CA-H1047L was a gift from Gordon Mills and Kenneth Scott (RRID: Addgene_116499). pHAGE-AKT1-E17K was a gift from Gordon Mills and Kenneth Scott (RRID: Addgene_116103).

### The Cancer Genome Atlas and the Clinical Proteomic Tumor Analysis Consortium human HNSCC dataset analysis

Level 3 mRNA sequencing (mRNA-seq) expression data and clinical information for The Cancer Genome Atlas (TCGA) HNSCC cohort, including 520 primary tumors and 44 matched normal tissues, were obtained from the Genomic Data Commons Data Portal as previously described (13). Expression data for normal esophageal mucosa were obtained from The Genotype-Tissue Expression (GTEx) Portal (GTEx Analysis Release V10). The *DESeq2* R package (RRID: SCR_000154) was used to calculate size factors and perform normalization to enhance the accuracy of the comparative analysis. The gene set enrichment analysis was performed using the default settings to evaluate the enrichment of differentially expressed genes in HNSCC compared with the matched normal tissue within the Hallmark gene sets. Overall survival (OS) analysis was conducted on the primary tumor subset using the *survival* R package (v3.7.0, RRID: SCR_021137) after stratifying patients by *SLC2A1* mRNA expression [log_2_(TPM+1)]. The optimal cutoff for *SLC2A1* expression was determined using the maximally selected rank statistic in the *survminer* R package (v0.5.0, RRID: SCR_021094). Kaplan–Meier curves were generated, and the log-rank test was used to compare OS between high- and low-*SLC2A1* expression groups. A Cox proportional hazards model was applied to calculate the HR. A 120-month follow-up threshold was applied. Quantitative proteomics data of human HNSCC tumors and matched normal tissue from the Clinical Proteomic Tumor Analysis Consortium was obtained from the LinkedOmicsKB portal, and normalized GLUT1 protein expression was compared (36).

### Quantification and statistical analysis

Statistical analyses were performed using StatPlus v5 (AnalystSoft, Inc.) GraphPad Prism 7.0 (RRID: SCR_002798), or R (version 4.4.2, RRID: SCR_001905). All data are expressed as mean ± SEM or median ± the IQR unless noted otherwise. The two-tailed Student *t* test, one-way ANOVA with multiple comparison *post hoc* test, Kruskal–Wallis nonparametric ANOVA, χ^2^ test, and Mann–Whitney *U* test were used for hypothesis testing wherever indicated, and *P* values of <0.05 were considered significant: ****, *P* < 0.0001; ***, *P* < 0.001; **, *P* < 0.01; *, *P* < 0.05.

### Data availability

The mRNA-seq and clinical cohort information referenced in this study are available in a public repository on TCGA website (www.cancer-genome.nih.gov). Normal tissue mRNA expression data are available on the GTEx Portal. Cell line expression data are available on the DepMap portal (DepMap Public 24Q2). The authors declare that all the other data supporting this study's findings are available within the article and from the corresponding authors upon request.

## Results

### GLUT1 is overexpressed in human patients with HNSCC

**Figure 1:**
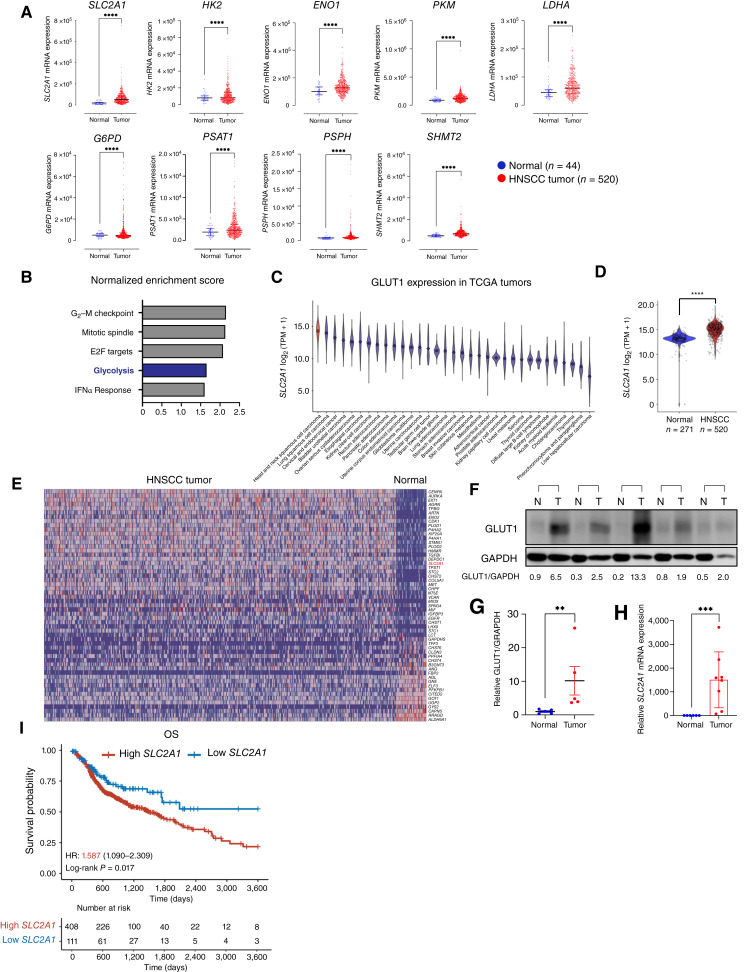
GLUT1 overexpression in TCGA HNSCC tumor samples. **A,** TCGA mRNA-seq analyses [normalized transcripts per million (TPM) mappable reads] of GLUT1 and metabolic genes associated with glycolysis, PPP, and *de novo* serine biosynthesis pathway in the patients of the TCGA HNSCC cohort (*n* = 520) and matched normal (*n* = 44) tissue samples. Each dot represents one patient. Error bars represent the median ± quartile range, Mann–Whitney *U* test. ****, *P* < 0.0001. **B,** Top five Hallmark gene sets enriched in patients of the TCGA HNSCC cohort are compared with matched normal tissue samples by gene set enrichment analysis. **C,** Violin plots depicting *SLC2A1* (GLUT1) mRNA expression in TCGA tumor samples stratified by lineage. Lineages are ordered by descending mean *SLC2A1* expression, and HNSCC is highlighted in red. **D,** Violin plot comparing *SLC2A1* (GLUT1) mRNA expression in TCGA HNSCC tumors with GTEx normal esophageal mucosa. Dots represent individual cell lines. Welch *t* test. ****, *P* < 0.0001. **E,** Heatmap indicating expression of genes enriched in the patients of the TCGA HNSCC cohort when compared with matched normal tissue samples. **F** and **G,** Immunoblot analysis and quantification of GLUT1 expression in human HNSCC tumor (*n* = 5) and matched normal head and neck epithelial (*n* = 5) tissue samples. **, *P* < 0.01. Error bars represent the median ± quartile range. Mann–Whitney *U* test. **H,***SLC2A1* mRNA expression from human HNSCC tumor samples (*n* = 8) and matched normal head and neck epithelial tissue samples (*n* = 7). Error bars represent the mean ± SEM. Mann–Whitney *U* test. ***, *P* < 0.001. **I,** Kaplan–Meier survival curves depicting OS of patients with HNSCC (*n* = 519) up to 120 months, stratified by *SLC2A1* mRNA expression. High- and low-*SLC2A1* expression groups were determined using the maximally selected rank statistic. A significant difference in OS between groups was assessed using the log-rank test, and a Cox proportional hazards model was applied to calculate the HR. *ENO1*, enolase 1; *G6PD*, glucose-6-phosphate dehydrogenase; *HK2*, hexokinase 2; *PKM*, pyruvate kinase M; *PSAT1*, phosphoserine aminotransferase 1; *PSPH*, phosphoserine phosphatase; *SHMT2*, serine hydroxymethyltransferase 2; *SLC2A1*, solute carrier family 2 member 1.

To query differences in gene expression that may imply metabolic alterations in HNSCC, we analyzed TCGA RNA-seq data from patients with HNSCC (*n* = 520) and matched normal tissue (*n* = 44) samples. We identified a robust upregulation of GLUT1 (*SLC2A1*), a key rate-limiting factor in the transport of glucose in cancer cells, and genes involved in central glucose metabolism (*HK2*, *ENO1*, *PKM*, and *LDHA*), PPP (*G6PD*), and *de novo* serine biosynthesis pathway (*PSAT1*, *PSPH*, and *SHMT2*; Fig. 1A). This differential gene expression in HNSCC tumor samples suggests potential alterations of metabolic pathways, including glucose metabolism. To further explore metabolic reprogramming in HNSCC, we analyzed metabolite abundance in Cancer Cell Line Encyclopedia cell lines obtained from the DepMap portal (DepMap Public 24Q2) and found glycolytic intermediates (F1P/F6P/G1P/G6P, DHAP, and G3P), lactate, PPP intermediate (R5P), and glutathione metabolism [glutathione (GSH) and glutathione disulfide] to be more abundant in HNSCC than in other cancer cell lines (Supplementary Fig. S1A).

Gene set enrichment analysis using the Hallmark gene sets in TCGA RNA-seq data comparing HNSCC tumor tissues with matched normal tissue samples identified glycolysis among the topmost significantly enriched gene sets in HNSCC (Fig. 1B; Supplementary Fig. S1B). Given that the elevated GLUT1 expression observed may simply be a feature of highly proliferative cells, we extended our analysis to compare HNSCC with other tumor and cancer cell line subtypes from TCGA and DepMap mRNA expression data, respectively. We found mean *SLC2A1* mRNA expression to be the highest in HNSCC among tumor subtypes (Fig. 1C) and the fourth highest among cancer cell line subtypes (Supplementary Fig. S1C). Furthermore, HNSCC exhibited significantly elevated *SLC2A1* mRNA expression compared with both normal esophageal mucosa (Fig. 1D) and pan-cancer cell lines (Supplementary Fig. S1D), and GLUT1 protein expression was observed to be higher in HNSCC tumors than in normal tissue (Supplementary Fig. S1E).

The analysis of normalized mRNA-seq gene expression profiles from HNSCC tumor and normal tissue samples identified the most highly differentially expressed genes (Fig. 1E). GLUT1 (*SLC2A1*) is among the most upregulated genes in HNSCC, along with genes involved in tumorigenicity and metabolism. Next, we sought to validate GLUT1 overexpression in human HNSCC tumor tissue samples. Consistent with TCGA data analyses, human HNSCC tumors have significantly higher mRNA and protein GLUT1 expression than matched normal tissues (Fig. 1F–H). HPV is a well-established etiologic and pathologic factor for HNSCC with HPV+ and HPV− tumors exhibiting distinct clinical and molecular features (37). Given these differences, we further stratified GLUT1 expression levels by HPV status to assess whether GLUT1 expression varies between HPV+ and HPV− in HNSCC tumor samples. GLUT1 expression is highly elevated regardless of the HPV status, but intriguingly, HPV− tumors show significantly higher GLUT1 expression than HPV+ tumors (Supplementary Fig. S1F).

We next investigated the prognostic significance of GLUT1 expression in primary tumors from the TCGA HNSCC cohort. Patients were stratified by *SLC2A1* mRNA expression, and Kaplan–Meier survival curves were generated to compare survival between expression groups. Our analysis shows that high GLUT1 expression is significantly associated with poorer OS (*P*_log-rank_ = 0.017) with a hazard ratio of 1.587 (Fig. 1I). Taken together, these results suggest that central carbon metabolism is altered in HNSCC and that enhanced glucose uptake and use may be differentially important for HNSCC progression.

### GLUT1 KO attenuates tumor growth in a 4NQO mouse model of HNSCC

**Figure 2:**
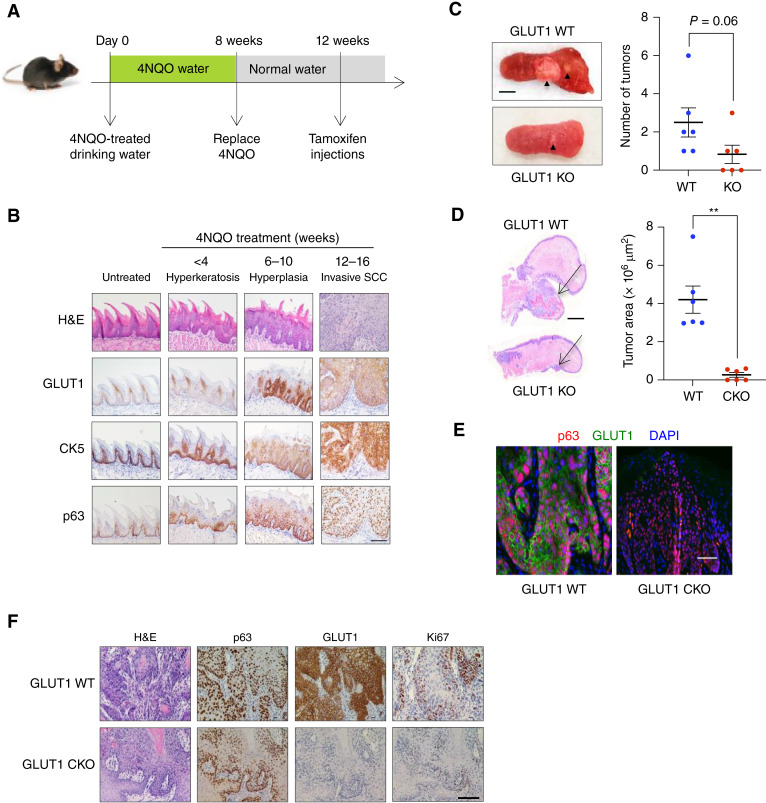
GLUT1 ablation attenuates tumorigenesis in 4NQO-induced mouse model of human HNSCC. **A,** Schematic showing timeline for treatment of mice with 4NQO. 4NQO was administered in drinking water for 8 weeks and replaced with normal drinking water for the remainder of the study. Tongues were collected every 2 weeks after the first exposure to 4NQO for analyses. For Cre-mediated GLUT1 ablation in basal epithelial cells, tamoxifen was injected (100 mg/kg i.p. daily for 5 consecutive days) 4 weeks after the final administration of 4NQO. Tumors were harvested 4 weeks after the final tamoxifen injection. **B,** Representative IHC analyses of GLUT1 expression in CK5^+^ and p63^+^ basal epithelial cells of untreated and 4NQO-treated mice. **C,** Representative images of tumor-bearing tongues from 4NQO-treated mice; black arrowheads depict squamous tongue tumors (left). Graph shows quantification of the number of visible tongue tumors identified in 4NQO-treated WT and Krt5^+^ basal epithelial–specific GLUT1 CKO mice (*n* = 6 for each group). Mann–Whitney *U* test. *P* = 0.06 (right). **D,** Representative H&E images of 4NQO-treated WT and CKO mouse tongues. Arrows depict 4NQO-induced squamous tumors (left). Graph shows quantification of 4NQO-induced tongue tumor area (μm^2^) in GLUT1 WT and CKO mice (*n* = 6 for each group). Mann–Whitney *U* test. **, *P* < 0.01 (right). **E,** Representative co-immunofluorescent staining of GLUT1 and p63 in 4NQO-induced squamous tongue tumors of GLUT1 WT and GLUT1 CKO mice. DAPI, 4',6-diamidino-2-phenylindole. **F,** Representative IHC analyses of p63, GLUT1, and Ki67 expression in 4NQO-induced squamous tongue tumors. All error bars represent mean ± SEM. All scale bars, 1 mm unless otherwise noted. H&E, hematoxylin and eosin; KO, knockout.

We next sought to assess whether GLUT1 overexpression in human HNSCC may be recapitulated in the well-characterized 4NQO-induced animal model of human oral SCC, a subset of HNSCC. 4NQO is a synthetic water-soluble compound that has been shown to cause DNA adduct formation and induce intracellular oxidative stress, thereby mimicking chronic tobacco smoke inhalation and excessive alcohol intake, which are major etiologic contributors to oncogenicity in HNSCC (31, 38, 39). Histologic analysis of the tongue tissue harvested from mice exposed to 4NQO in drinking water (100 μg/mL) for 8 weeks revealed a sequential progression to SCC, starting from hyperplasia followed by dysplasia and invasive SCC (Fig. 2A and B). IHC analysis confirmed the expression of squamous lineage markers, keratin 5 (Krt5 and CK5) and p63 in 4NQO-induced tumors (Fig. 2B). Tongues collected from mice treated with 4NQO for 4 weeks exhibited hyperkeratosis in the mucosal epithelium, which progressed from moderate to severe hyperplasia observed in mouse tongues collected 6 to 10 weeks after exposure to 4NQO (Fig. 2B). Severe dysplastic lesions and incidences of invasive SCC were observed in mouse tongues collected 12 to 16 weeks after the initial exposure to 4NQO (Fig. 2B). Notably, during the development of premalignant lesions, we observed a dramatic increase in GLUT1 expression in basal epithelial cells, suggesting that GLUT1 upregulation may be an early event during the progression to malignancy (Fig. 2B).

To investigate whether genetic ablation of GLUT1 in the Krt5+ basal progenitor cells—potential cells of origin of SCC—might effectively inhibit or halt squamous cancer progression after tumor initiation, we established a tamoxifen-inducible knockout mouse model (*Krt5:creER*; *LSL-dTomato*; *T*rp*53*^*+/−*^; *Slc2a1*^*loxP/loxP*^; detailed in “Materials and Methods”). This model allowed us to evaluate the effect of basal epithelial cell–specific GLUT1 ablation in HNSCC. *Krt5:creER*; *LSL-dTomato*; *Trp53*^*+/−*^; *Slc2a1*^*loxP/loxP*^ [GLUT1 conditional knockout (CKO)] and *Krt5:creER*; *LSL-dTomato*; *T*rp*53*^*+/−*^; *Slc2a1*^*+/+*^ [GLUT1 wild-type (WT)] mice were administered 4NQO-treated drinking water for 8 weeks to induce SCC tumors (Fig. 2A). At 16 weeks after the initial exposure to 4NQO, all GLUT1 WT mice developed visible tumors on the dorsal or ventral surface of their tongues (arrowheads in Fig. 2C depict two visible tumors), whereas 50% of the GLUT1 CKO mice showed no visible tumors. The remaining GLUT1 CKO mice had significantly smaller tumors than the GLUT1 WT mice (Fig. 2C and D). Co-immunofluorescent staining, as well as IHC analysis on serial sections, validated the specific deletion of GLUT1 in Krt5+ basal epithelial cells, including p63^+^ squamous cancer cells in the GLUT1 CKO mice (Fig. 2E). Furthermore, GLUT1 CKO tumor cells exhibited markedly decreased Ki67 nuclear staining, indicating the importance of GLUT1 in SCC cell proliferation (Fig. 2F). These results suggest that GLUT1 overexpression in the basal progenitor cells is a crucial and early step necessary for malignant squamous cancer progression and proliferation.

### GLUT1 inhibition enhances cytotoxic oxidative stress in HNSCC cells

**Figure 3:**
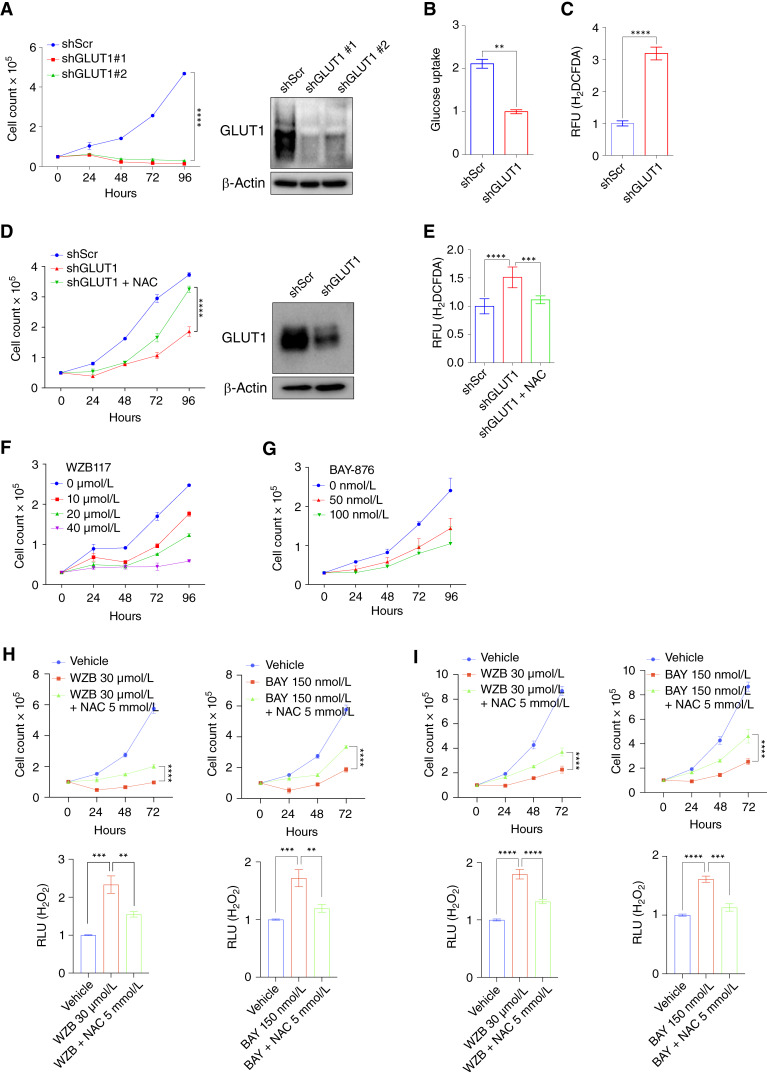
GLUT1 inhibition enhances cytotoxic oxidative stress in human HNSCC cell lines. **A,***In vitro* proliferation and immunoblot analyses of shScramble (shScr) and shRNA-mediated KD of GLUT1 (shGLUT1) in HNSCC cell line FaDu (*n* = 4 for each group). Two-way ANOVA. ****, *P* < 0.0001 (left). Immunoblot validation of shRNA-mediated KD of GLUT1 in FaDu cells (right). **B,** Relative glucose uptake and (**C**) intracellular ROS measurement in shScr and shGLUT1 FaDu cells (*n* = 3 for each group). Two-tailed *t* test. ***, *P* < 0.001; **, *P* < 0.01. **D,***In vitro* proliferation of shScr, shGLUT1, and shGLUT1 + NAC (5 mmol/L) in HNSCC cell line SCC-61 (*n* = 4 for each group). Two-way ANOVA. ****, *P* < 0.0001 (left). Immunoblot validation shRNA-mediated KD of GLUT1 in SCC-61 cells (right). **E,** Intracellular ROS measurement of shScr, shGLUT1, and shGLUT1 + NAC (5 mmol/L) in SCC-61 cells (*n* = 3 for each group). Two-tailed *t* test. ****, *P* < 0.0001; ***, *P* < 0.001. **F** and **G,***In vitro* proliferation of FaDu cells treated with GLUT1 inhibitors, WZB117 (0–40 μmol/L) or BAY-876 (0–100 nmol/L; *n* = 3 for each group). **H** and **I,***In vitro* proliferation of FaDu (**H**) or SCC-61 (**I**) treated with WZB117 (30 μmol/L) or BAY-876 (150 nmol/L) with or without 5 mmol/L NAC treatment (*n* = 6 for each group). Two-way ANOVA. ****, *P* < 0.0001; **, *P* < 0.01; *, *P* < 0.05 (top). Intracellular H_2_O_2_ measurement of FaDu (**H**) or SCC-61 (**I**) cells treated with WZB117 (30 μmol/L) or BAY-876 (150 nmol/L) with or without 5 mmol/L NAC treatment (*n* = 6 for each group). Two-tailed *t* test. ****, *P* < 0.0001; ***, *P* < 0.001; **, *P* < 0.01 (bottom). All error bars represent mean ± SEM unless otherwise noted. BAY, BAY-876; RFU, relative fluorescent unit; RLU, relative light unit; WZB, WZB117.

To elucidate the functional role of GLUT1 in human HNSCC, we used both genetic and pharmacologic approaches to inhibit GLUT1 activity. GLUT1 knockdown (GLUT1 KD) via shRNA in the FaDu human HNSCC cell line dramatically impaired cell proliferation (Fig. 3A). Additionally, GLUT1 KD reduced glucose uptake, accompanied by increased oxidative stress in FaDu cells (Fig. 3B and C). These results align with our previous reports and indicate that HNSCC cells rely on GLUT1-mediated glucose uptake to maintain antioxidant capacity (13, 14). To ask whether antioxidant treatment might be sufficient to restore growth upon GLUT1 KD, we treated the HNSCC cell line SCC-61 expressing *SLC2A1* shRNA with N-acetylcysteine (NAC). We found that NAC treatment substantially restored proliferation and reduced intracellular ROS, suggesting that GLUT1-mediated glucose uptake supports redox homeostasis to maintain cell proliferation (Fig. 3D and E).

We further perturbed glucose influx by pharmacologically inhibiting GLUT1 using well-characterized, irreversible GLUT1 inhibitors WZB117 and BAY-876 (32, 35). Treatment with these inhibitors resulted in a dose-dependent decrease in FaDu and SCC-61 cell proliferation (Fig. 3F and G). Consistent with the prior observation that antioxidant administration was sufficient to overcome shGLUT1-mediated growth inhibition, NAC treatment partially rescued proliferation and substantially reduced oxidative stress in FaDu and SCC-61 cells after GLUT1 inhibition (Fig. 3H and I).

**Figure 4:**
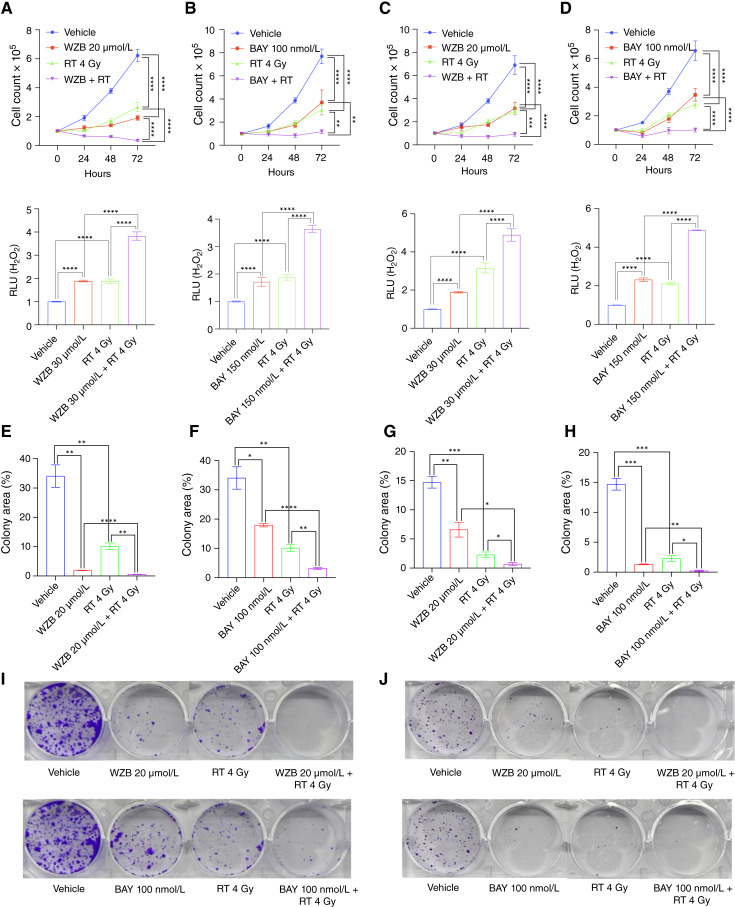
GLUT1 inhibition potentiates oxidative cytotoxicity of radiation in human HNSCC cell lines. **A** and **B,***In vitro* proliferation (top) and intracellular H_2_O_2_ levels (bottom) of FaDu cells treated with WZB117 (20 μmol/L; **A**) or BAY-876 (100 nmol/L; **B**) with or without radiotherapy (RT, 4 Gy; *n* = 6 for each group). Two-way ANOVA (top) or two-tailed *t* test (bottom). ****, *P* < 0.0001; **, *P* < 0.01. **C** and **D,***In vitro* proliferation (top) and intracellular H_2_O_2_ levels (bottom) of SCC-61 cells treated with WZB117 (20 μmol/L; **C**) or BAY-876 (100 nmol/L; **D**) with or without radiation (RT, 4 Gy; *n* = 6 for each group). Two-way ANOVA (top) or two-tailed *t* test (bottom). ****, *P* < 0.0001; ***, *P* < 0.001. **E–J,** Colony formation of HNSCC cells FaDu (**E**, **F**, and **I**) and Detroit 562 (**G**, **H**, and **J**) treated with WZB117 (20 μmol/L) or BAY-876 (100 nmol/L) with or without RT (4 Gy). Colonies were stained with crystal violet, and the colony area was measured using ImageJ (*n* = 3). Two-tailed *t* test. ****, *P* < 0.0001; ***, *P* < 0.001; **, *P* < 0.01; *, *P* < 0.05. BAY, BAY-876; RLU, relative light unit; WZB, WZB117.

Having established that GLUT1 inhibition induces oxidative stress, we next examined whether targeting GLUT1 might potentiate the oxidative cytotoxic effects of ionizing radiation, a first-line treatment for HNSCC (40). Treatment of FaDu and SCC-61 cells with a combination of GLUT1 inhibitors WZB117 (20 μmol/L) or BAY-876 (100 nmol/L) and radiation (4 Gy) significantly precipitated oxidative stress and produced an additive antiproliferative effect (Fig. 4A–D). Furthermore, to establish the radiosensitizing effect of GLUT1 inhibition, we performed clonogenic assays after the exposure of HNSCC cells to GLUT1 inhibitor, ionizing radiation, or their combination (Fig. 4E–J). The combination treatment led to a significant reduction in the size and number of colonies formed compared with both the untreated control and individual treatments. Collectively, these findings implicate GLUT1-mediated glucose uptake in maintaining antioxidant capacity and suggest that targeting glucose metabolism may enhance the cytotoxic effect of radiation.

### Combination of GLUT1 inhibition and ascorbate suppresses HNSCC tumor growth

**Figure 5:**
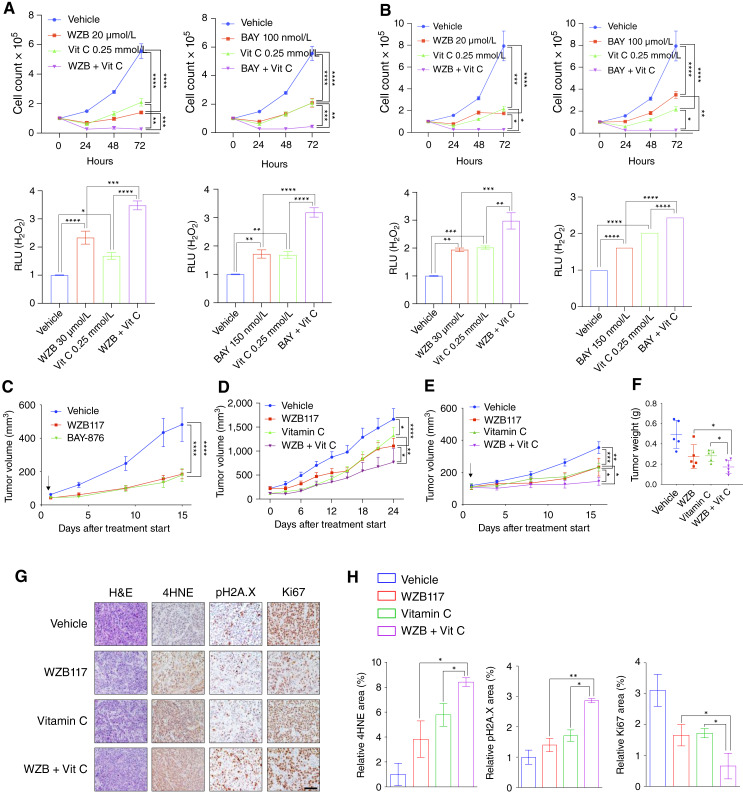
Combination of GLUT1 inhibition and ascorbate suppresses HNSCC tumor growth. **A** and **B,***In vitro* proliferation (top) and intracellular H_2_O_2_ levels (bottom) of FaDu (**A**) or SCC-61 (**B**) cells treated with WZB117 (20 μmol/L) or BAY-876 (100 nmol/L) and vitamin C (0.25 mmol/L) or combination of GLUT1 inhibitor and vitamin C (*n* = 5 for each group). Two-way ANOVA (top) or two-tailed *t* test (bottom). ****, *P* < 0.0001; ***, *P* < 0.001; **, *P* < 0.01; *, *P* < 0.05. **C,***In vivo* tumor growth of FaDu xenografts treated with vehicle PBS/DMSO, WZB117 (20 mg/kg i.p. daily), or BAY-876 (4.5 mg/kg of corn oil/DMSO p.o. daily; *n* = 6 for each group). Two-way ANOVA. ****, *P* < 0.0001. **D,***In vivo* tumor growth of FaDu xenograft tumors treated with vehicle PBS/DMSO (*n* = 16), WZB117 (20 mg/kg i.p. daily; *n* = 16), vitamin C (4 g/kg i.p. daily; *n* = 7), and a combination of WZB117 and vitamin C (*n* = 7). Two-way ANOVA. ****, *P* < 0.0001; **, *P* < 0.01; *, *P* < 0.05. **E–G,***In vivo* tumor growth of SCC-61 xenografts (**E**), tumor weights (**F**), IHC analysis of 4HNE, Ki67, and pH2A.X (**G**) in SCC-61 xenograft tumors treated with vehicle PBS/DMSO (*n* = 6), WZB117 (20 mg/kg i.p. daily; *n* = 5), vitamin C (4 g/kg i.p. daily; *n* = 5), or combination of WZB117 and vitamin C (*n* = 6). Two-way ANOVA. **, *P* < 0.01; *, *P* < 0.05. Scale bars, 1 mm. **H,** IHC analysis quantification of % area of 4HNE (lipid peroxidation marker), pH2A.X (DNA damage marker), and Ki67 (nuclear/cell proliferation marker) in xenografted tumors. Four to six images of each tumor were captured and analyzed for quantification. Two-tailed *t* test. *, *P* < 0.05. All error bars represent mean ± SEM. BAY, BAY-876; H&E, hematoxylin and eosin; Vit C, vitamin C; RLU, relative light unit; WZB, WZB117.

Building on our observation that GLUT1 inhibition can sensitize HNSCC cells to oxidative stress, we next explored whether co-treatment with a pro-oxidant could further increase cytotoxicity. Pharmacologic concentrations of vitamin C have been shown to function as a pro-oxidant by depleting glutathione after being taken up in its oxidized form dehydroascorbate and being reduced at the expense of GSH, NADPH, and thioredoxin, thereby exerting cytotoxic effects on various cancer types, including squamous cancers (41, 42). Indeed, treating HNSCC cells (FaDu and SCC-61) with a combination of GLUT1 inhibitors (WZB117, 20 μmol/L, or BAY-876, 100 nmol/L) and vitamin C (0.25 mmol/L) dramatically reduced cellular proliferation compared with either monotherapy (Fig. 5A and B). The combination of GLUT1 inhibitors and vitamin C treatment elicited higher intracellular H_2_O_2_ levels, suggesting that ROS contributes to the reduced viability (Fig. 5A and B).

To evaluate the *in vivo* effects of GLUT1 inhibition in HNSCC, we first treated SCID mice bearing FaDu xenograft tumors with either WZB117 (10 mg/kg daily) or BAY-876 (4.5 mg/kg daily) for 2 weeks and observed that both inhibitors significantly reduced tumor growth compared with vehicle-treated controls (Fig. 5C; Supplementary Fig. S2A). We next assessed whether combining WZB117 with vitamin C (4 g/kg daily) would further enhance tumor suppression in both FaDu (Fig. 5D; Supplementary Fig. S2B) and SCC-61 (Fig. 5E; Supplementary Fig. S2C) xenograft models. Indeed, mice receiving WZB117 with vitamin C exhibited significantly diminished tumor volumes and reduced tumor weights (Fig. 5F) compared with either monotherapy group in both models. IHC analysis revealed increased 4HNE (indicative of lipid peroxidation) and γ-H2AX (a marker of DNA double-strand breaks) staining in SCC-61 tumors in the combined treatment group compares with single-agent treatments (Fig. 5G and H). These results implicate the ability of combined GLUT1 inhibition and ascorbate administration to more significantly disrupt redox homeostasis and also impede HNSCC tumor progression *in vivo*.

### Auranofin induces oxidative stress and cytotoxicity in HNSCC

**Figure 6:**
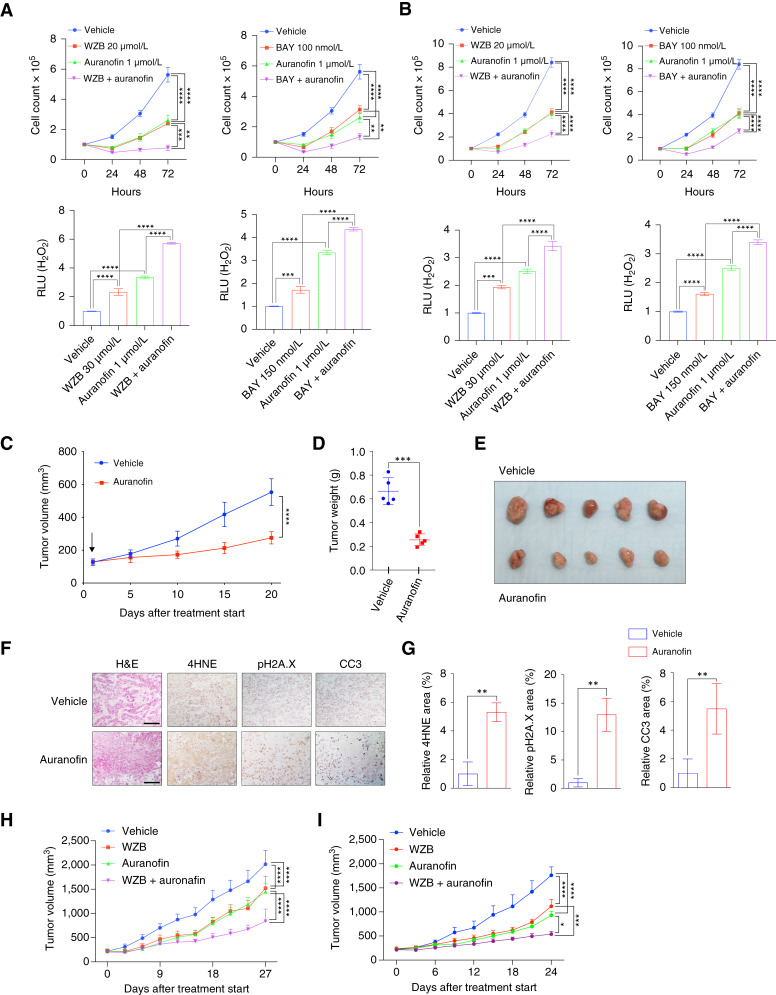
Auranofin induces cytotoxicity as a pro-oxidant in HNSCC. **A** and **B,***In vitro* proliferation (top) and intracellular H_2_O_2_ levels (bottom) of FaDu (**A**) or SCC-61 (**B**) cells treated with vehicle, WZB117 (20 μmol/L), auranofin (1 μmol/L), or combination of WZB117 and auranofin (*n* = 6 for each group). Two-way ANOVA (top) or two-tailed *t* test (bottom). ****, *P* < 0.0001; ***, *P* < 0.001; **, *P* < 0.01. **C–F,***In vivo* tumor growth (**C**), tumor weights (**D**), images of individual tumors (**E**), and IHC analysis of GLUT1, pH2A.X, 4HNE, and CC3 (**F**) in SCC-61 xenograft tumors treated with vehicle PBS/DMSO (*n* = 6) or auranofin (10 mg/kg i.p. daily; *n* = 6). Two-way ANOVA for tumor growth (**C**) and two-tailed *t* test for tumor weight (**D**). ****, *P* < 0.0001; ***, *P* < 0.001. **G,** IHC analysis quantification of % area of 4HNE (lipid peroxidation marker), pH2A.X (DNA damage marker), and CC3 (apoptotic cell marker) in xenografted tumors (*n* = 6 for each group). Four to six images of each tumor were captured and analyzed for quantification. Two-tailed *t* test. **, *P* < 0.01. **H,***In vivo* tumor growth of FaDu xenograft tumors treated with vehicle PBS/DMSO (*n* = 16), WZB117 (20 mg/kg i.p. daily; *n* = 16), auranofin (10 mg/kg i.p. daily; *n* = 15), or combination of WZB117 and auranofin (*n* = 12). Two-way ANOVA. ****, *P* < 0.0001; ***, *P* < 0.001; **, *P* < 0.01. **I,***In vivo* tumor growth of SCC-61 xenograft tumors treated with vehicle PBS/DMSO (*n* = 6), WZB117 (20 mg/kg i.p. daily; *n* = 7), auranofin (10 mg/kg i.p. daily; *n* = 7), or combination of WZB117 and auranofin (*n* = 7). Two-way ANOVA. ****, *P* < 0.0001; ***, *P* < 0.001; **, *P* < 0.01. All error bars represent mean ± SEM unless otherwise noted. Scale bars, 1 mm. BAY, BAY-876; H&E, hematoxylin and eosin; RLU, relative light unit; WZB, WZB117.

To explore potential drug candidates that exploit redox vulnerabilities in HNSCC and may be rapidly translatable, we used the Drug Repurposing Hub (Broad Institute, https://clue.io/repurposing) to search for FDA-approved drugs targeting key antioxidant enzymes. We selected auranofin, an FDA-approved treatment for rheumatoid arthritis, which has been shown to inhibit thioredoxin reductase 1, an antioxidant enzyme that acts on electron cycling in the NADPH/GSH electron coupling system (43–45). We hypothesized that disrupting thioredoxin reductase activity with auranofin would compromise the cellular redox balance in HNSCC. Indeed, treating SCC-61 cells with auranofin decreased proliferation and increased oxidative stress (Supplementary Fig. S3A and S3B). Moreover, combining auranofin with GLUT1 inhibitors elicited additive antiproliferative activities and heightened oxidative stress in both FaDu and SCC-61 cell lines (Fig. 6A and B).

Next, we evaluated the *in vivo* efficacy of auranofin in an SCC-61 xenograft model. Intraperitoneal injection of auranofin (10 mg/kg daily) significantly reduced SCC-61 xenograft tumor growth compared with vehicle-treated controls (Fig. 6C–E; Supplementary Fig. S2D). IHC analysis revealed increased levels of γ-H2AX (a marker of DNA double-strand breaks), 4HNE (lipid peroxidation), and CC3 (apoptotic cell death) in auranofin-treated tumors (Fig. 6F and G).

We further tested whether combining auranofin with GLUT1 inhibition might be tolerated *in vivo* and elicit enhanced tumor suppression compared with either agent alone. Indeed, co-treatment with WZB117 and auranofin yielded greater antitumor activity than that of monotherapies (Fig. 6H and I; Supplementary Fig. S2E and S2F). Collectively, these findings support auranofin as a potentially translatable therapy that exploits redox imbalances and can be combined with other interventions to achieve more robust control of HNSCC.

### Insulin signaling promotes glucose-mediated antioxidative capacities in HNSCC

**Figure 7:**
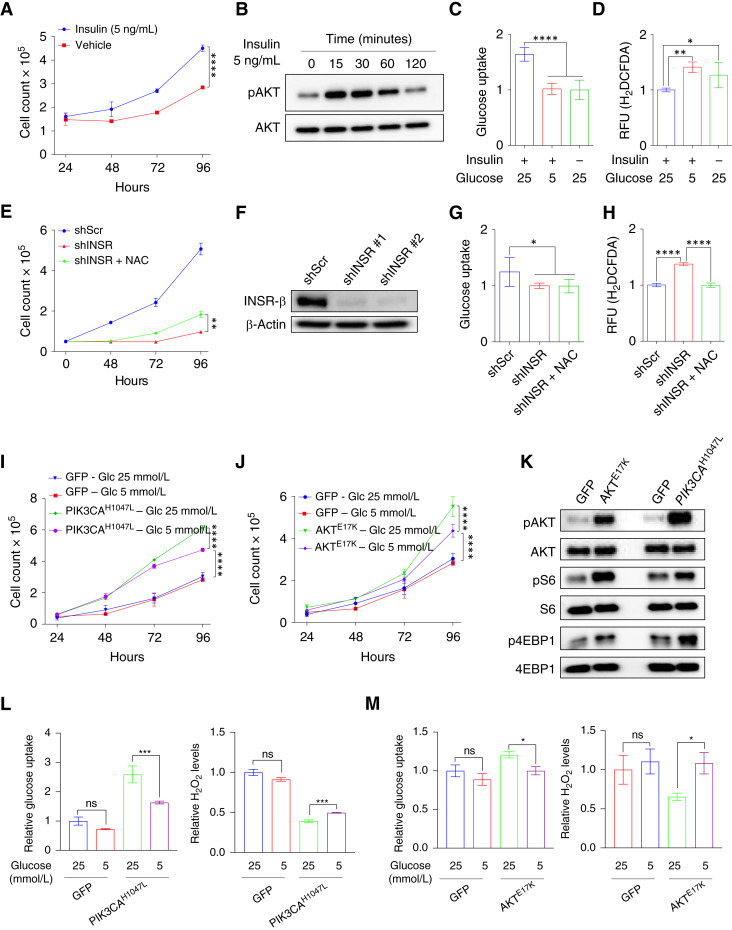
Insulin signaling promotes glucose-mediated antioxidative capacities in HNSCC. **A,***In vitro* proliferation of FaDu cells in 25 mmol/L glucose media treated with or without 5 ng/mL insulin (*n* = 4 for each group). Two-way ANOVA. ****, *P* < 0.0001. **B,** Immunoblot analysis of pAKT and total AKT expression in FaDu cells treated with 20 ng/mL insulin over a time period of 0–120 minutes. **C** and **D,** Relative glucose uptake (**C**) and intracellular ROS measurement (**D**) in FaDu cells treated with 20 ng/mL insulin in 25 or 5 mmol/L glucose conditions (*n* = 3 for each group). Two-tailed *t* test. ****, *P* < 0.0001; **, *P* < 0.01; *, *P* < 0.05. **E,***In vitro* proliferation of shScr, shINSR, or shINSR + 5 mmol/L NAC FaDu cells (*n* = 4 for each group). Two-way ANOVA. **, *P* < 0.01. **F,** Immunoblot analyses of shScr and shINSR FaDu cells. β-Actin was used as the loading control. **G** and **H,** Relative glucose uptake (**G**) and intracellular ROS levels (**H**) in shScr and shINSR FaDu cells (*n* = 3). Two-tailed *t* test. ****, *P* < 0.0001; *, *P* < 0.05. **I** and **J,***In vitro* proliferation of shScr, oncogenic mutant AKT^E17K^ and PIK3CA^H1047L^ expressing FaDu cells grown in 5 or 25 mmol/L glucose media (*n* = 4 for each group). Two-way ANOVA. ****, *P* < 0.0001. **K,** Immunoblot analysis of pAKT, AKT, pS6, S6, p4EBP1, and 4EBP1 expression in shScr and mutant AKT^E17K^ and PIK3CA^H1047L^ FaDu cells. **L,** Relative cellular glucose uptake and intracellular H_2_O_2_ levels in FaDu PIK3CA^H1047L^ mutant cells cultured in 5 or 25 mmol/L glucose (*n* = 3 for each group). Two-tailed *t* test. ***, *P* < 0.001. **M,** Relative cellular glucose uptake and intracellular H_2_O_2_ levels in FaDu AKT^E17K^ mutant cells cultured in 5 or 25 mmol/L glucose (*n* = 3 for each group). Two-tailed *t* test. *, *P* < 0.05. All error bars represent mean ± SEM unless otherwise noted. Glc, glucose; RFU, relative fluorescence unit; ns, not significant.

We next sought to determine the contribution of insulin signaling to glucose uptake and redox homeostasis in the context of the GLUT1 overexpression that is observed in HNSCC. To first investigate the effect of insulin signaling on glucose-dependent redox homeostasis, we first exposed human HNSCC cells to near-physiologic levels of exogenous insulin under serum-free conditions. Insulin treatment promoted proliferation and AKT activation, highlighting, perhaps not surprisingly, that insulin acts as a significant growth factor (Fig. 7A and B). Consistent with a link between insulin signaling and glucose uptake, insulin treatment also reduced intracellular H_2_O_2_ levels but only when glucose was abundant (Fig. 7C and D). Under lower glucose conditions, insulin failed to suppress H_2_O_2_ levels, suggesting that insulin-driven effects on redox homeostasis are depend on adequate extracellular glucose.

To directly assess the requirement of insulin signaling for maintaining antioxidant capacity, we performed shRNA-mediated KD of the insulin receptor (IR/INSR). INSR KD reduced glucose uptake, elevated intracellular ROS, and inhibited HNSCC cell proliferation, even in high-glucose media (Fig. 7E–H). Notably, antioxidant treatment provided only a partial rescue of cell growth, implying that enhanced oxidative stress contributes to, but does not fully account for, the loss of proliferation upon INSR ablation (Fig. 7G and H). Nevertheless, these findings suggest that INSR signaling is necessary to maintain glucose uptake and redox homeostasis despite the overexpression of GLUT1.

We next introduced constitutively active PI3K (PIK3CA^H1047L^) or AKT (AKT^E17K^) mutants to examine whether oncogenic PI3K–AKT signaling could enhance growth and redox control under varying glucose conditions. Indeed, constitutively active PI3K signaling drove greater proliferation under both glucose-replete and glucose-limiting conditions than GFP control cells (Fig. 7I–K). However, lowering glucose to 5 mmol/L slightly but significantly restrained growth of the mutants, presumably by reducing glucose uptake and elevating ROS (Fig. 7L and M). Of note, GFP-expressing control cells were unaffected by this glucose restriction, suggesting that constitutively active PI3K–AKT signaling raises metabolic demands that rely on ample glucose. Collectively, these results suggest that PI3K–AKT signaling indeed drives glucose uptake and elevates antioxidant capacity even under glucose-limiting conditions.

### Dysregulated insulin signaling reduces HNSCC tumor growth

**Figure 8:**
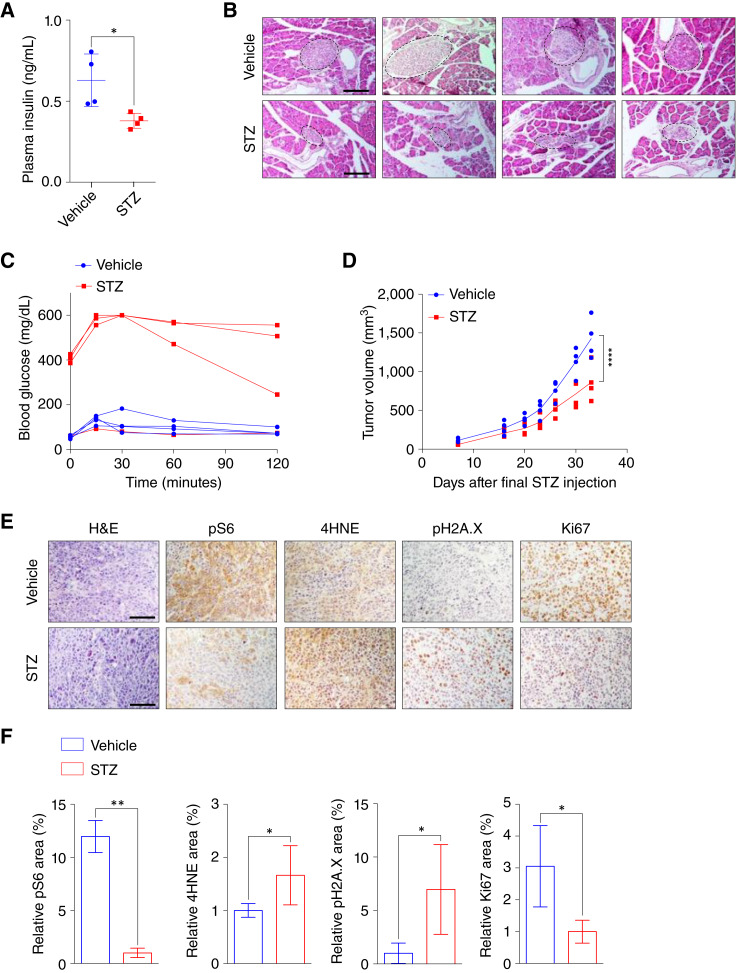
Dysregulated insulin signaling reduces HNSCC tumor growth. **A,** Plasma insulin concentration of vehicle- or STZ-treated mice (40 mg/kg i.p. for 5 consecutive days; *n* = 4 for each group). Two-tailed *t* test. *, *P* < 0.05. **B,** Representative H&E staining of pancreatic tissues collected from vehicle- or STZ-treated mice. Dashed lines denote individual pancreatic islets. Scale bars, 1 mm. **C,** Intraperitoneal glucose tolerance test of vehicle- and STZ-treated mice (*n* = 4 for each group). **D,***In vivo* tumor growth of FaDu xenograft tumors in vehicle-treated (*n* = 4) and STZ-treated (*n* = 4) mice. Two-way ANOVA. ****, *P* < 0.0001. **E,** H&E and IHC analyses of pH2A.X, 4HNE, and Ki67 in FaDu xenograft tumors collected from vehicle- and STZ-treated mice. Scale bars, 1 mm. **F,** IHC analysis quantification of % area of 4HNE (lipid peroxidation marker), pH2A.X (DNA damage marker), and Ki67 (nuclear/cell proliferation marker) in xenografted tumors. Four to six images of each tumor were captured and analyzed for quantification (*n* = 4 for each group). Two-tailed *t* test, **, *P* < 0.01; *, *P* < 0.05. All error bars represent mean ± SEM unless otherwise noted. H&E, hematoxylin and eosin.

To determine whether reduced insulin signaling affects HNSCC tumor growth *in vivo*, we used a STZ-induced type I diabetes mouse model (46). STZ depletes pancreatic β cells, thereby lowering systemic insulin levels (Fig. 8A and B) and severely disrupting glucose homeostasis as indicated by sustained hyperglycemia after an intraperitoneally injected glucose load (Fig. 8C). Despite this marked hyperglycemia, HNSCC tumors in STZ-treated mice grew significantly more slowly than those in control animals (Fig. 8D). IHC staining showed decreased pS6 and Ki67, indicative of reduced growth signaling and proliferation, along with elevated 4HNE and γ-H2AX, reflecting increased oxidative stress and DNA damage in tumors from STZ-treated mice (Fig. 8E and F). These findings suggest that insulin, rather than simply abundant extracellular glucose, is necessary for sustaining tumor growth in HNSCC, likely by supporting glucose uptake, mitigating oxidative stress, and maintaining growth signaling pathways. Although intratumoral glucose flux was not directly measured, elevated oxidative stress and reduced proliferation in tumors from STZ-treated mice align with our *in vitro* findings that insulin signaling is necessary for glucose uptake and redox homeostasis, thereby highlighting the role of insulin in HNSCC progression.

## Discussion

Despite advances in treatment modalities, including in systemic therapies such as cetuximab and immune checkpoint inhibitors, which have improved outcomes in patients with HNSCC, HNSCC continues to pose a significant clinical challenge with substantial morbidity and mortality (1). The diverse anatomic origins and etiology of HNSCC contribute to considerable molecular heterogeneity, complicating therapeutic decisions and limiting the success of targeted approaches (47, 48). Surgery, chemotherapy, and radiotherapy remain the mainstays of treatment; however, high rates of relapse with locoregional disease and distant metastases underscore the need to identify and target more fundamental drivers of HNSCC that, when combined with existing therapies, may improve disease control and patient survival.

Our findings highlight GLUT1-mediated glucose influx and insulin signaling as critical for HNSCC progression and survival, in part by sustaining redox homeostasis. The initial analysis of a diverse set of tumor samples from patients with HNSCC spanning various anatomic origins (pharyngeal, laryngeal, and other oral cancers) in TCGA data unveiled marked GLUT1 overexpression. We recapitulated this key observation using the mouse model of human HNSCC induced by 4NQO, which mimics the pro-oncogenic effects of cigarette smoking or alcohol consumption by promoting oxidative cellular and DNA damage in squamous epithelial cells of the tongue and esophagus. Notably, GLUT1 upregulation in premalignant lesions suggests that squamous epithelium may rely on glucose-derived antioxidant capacity to manage carcinogen-induced oxidative stress, a necessary adaptation sustained during tumorigenesis. Moreover, genetic ablation of GLUT1 reduced both the incidence and size of 4NQO-induced tumors, supporting its essential role in malignant progression. Ultimately, the early upregulation and functional necessity of GLUT1 raise the possibility that it could serve as both a diagnostic marker and prophylactic target in HNSCC. In addition, both genetic ablation and pharmacologic inhibition of GLUT1 reduced HNSCC cell growth *in vitro* and *in vivo*. Although disrupting glucose uptake undoubtedly triggers pleiotropic antiproliferative effects, our results suggest that cytotoxicity is mediated in part by GLUT1 inhibition tipping the balance toward oxidative stress, a condition that can be further accentuated by the coadministration of pro-oxidants.

Given the profound influence of insulin signaling on central carbon metabolism, we sought to elucidate its role in HNSCC progression. Although clinical and epidemiologic studies have linked diabetes with increased HNSCC incidence and poorer prognoses, the specific connection between insulin signaling and glucose-fueled redox metabolism in HNSCC remains largely unexplored (49–51). Building upon our findings that a ketogenic diet reduced lung squamous cancer progression by restricting glucose availability and indirectly suppressing insulin signaling, our current study demonstrates that insulin–PI3K signaling enhances antioxidant capacity and that lowering glucose attenuates this effect (14). Notably, our data suggest that GLUT1 overexpression alone is insufficient to maintain optimal glucose uptake and redox homeostasis; rather, intact insulin signaling is required. This may be partly explained by AKT-mediated suppression of TXNIP, which normally promotes GLUT1 internalization, and by broader transcriptional and post-translational regulation of metabolic enzymes by the PI3K–AKT pathway (30, 52, 53). *In vitro*, insulin receptor KD significantly reduced proliferation, an effect that was only partially rescued by the antioxidant NAC, indicating that oxidative stress contributes to, but does not fully account for, the observed growth inhibition. Furthermore, in an STZ-induced hypoinsulinemic mouse model, HNSCC tumor growth was significantly impaired and accompanied by increased intratumoral oxidative stress despite systemic hyperglycemia. This suggests that the physiologic levels of insulin are critical to sustain tumor growth and that increased glucose availability alone cannot compensate. In the context of our broader finding, it is plausible that reduced insulin signaling limits the effective uptake and use of available glucose, thereby compromising antioxidant capacity and contributing to oxidative stress. Collectively, these observations underscore the essential role of intact insulin signaling in channeling glucose into metabolic and antioxidative pathways that support HNSCC progression and suggest that targeting this axis may offer a novel therapeutic strategy to counteract the protumorigenic effects of PI3K signaling.

New therapeutic modalities, including targeted therapies and immunotherapy, have shown promise in improving patient outcomes while reducing treatment-associated toxicity (3, 7, 54–56). Notably, molecular targets like EGFR, VEGF, and PI3K have been explored for HNSCC therapy, yet have yielded mixed outcomes (57–59). Our data advocate for an alternative strategy: targeting redox homeostasis by precipitating oxidative stress. In this regard, pro-oxidant agents—such as high-dose intravenous vitamin C and auranofin, an FDA-approved drug for rheumatoid arthritis that primarily inhibits thioredoxin reductase—may serve as effective radiosensitizers or chemosensitizers by further destabilizing the cellular redox equilibrium. Given that vitamin C, in its oxidized form dehydroascorbate, is primarily transported into cells via GLUT1, it is possible that vitamin C may more acutely disrupt redox in HNSCC cells (42, 60). Moreover, both our preclinical findings and ongoing clinical trials in ovarian cancer (NCT03456700), chronic lymphocytic leukemia (NCT01419691), and lung cancer (NCT01737502) support the potential efficacy of auranofin against HNSCC. Collectively, these observations motivate further investigation into potentially rapidly translatable combination therapies that exploit redox perturbation to enhance the cytotoxic effect of conventional treatments.

In summary, although metabolic alterations are recognized as key drivers of tumorigenesis across aggressive cancers, our study highlights a promising therapeutic strategy for HNSCC by targeting its metabolic and redox vulnerabilities. Although concerns have been raised about GLUT1 inhibition due to the expression of GLUT1 in critical normal tissues such as the blood–brain barrier, heart, and erythrocytes, our work and that of others have not observed significant cerebral or hematologic toxicity in animal models (13, 61). Ultimately, these findings demonstrate the potential of pro-oxidant therapies to destabilize tumor redox balance and warrant further preclinical and clinical evaluation to improve outcomes in patients with HNSCC.

## Supplementary Material

Figure S1 Supplementary Figure 1 and Legend

Figure S2 Supplementary Figure 2 and legend

Figure S3 Supplementary Figure 3 and legend
