# Supplementary material for: Targeting the Glucose–Insulin Link in Head and Neck Squamous Cell Carcinoma Induces Cytotoxic Oxidative Stress and Inhibits Cancer Growth
Source: Cancer Res Commun. 2025 Jun 6;5(6):921–38. doi: 10.1158/2767-9764.CRC-23-0506 (PMC12141995; doi:10.1158/2767-9764.CRC-23-0506)
Supplement: Figure S1 — Supplementary Figure 1 and Legend [file crc-23-0506_figure_s1_suppsf1.pptx]

## Slide 1
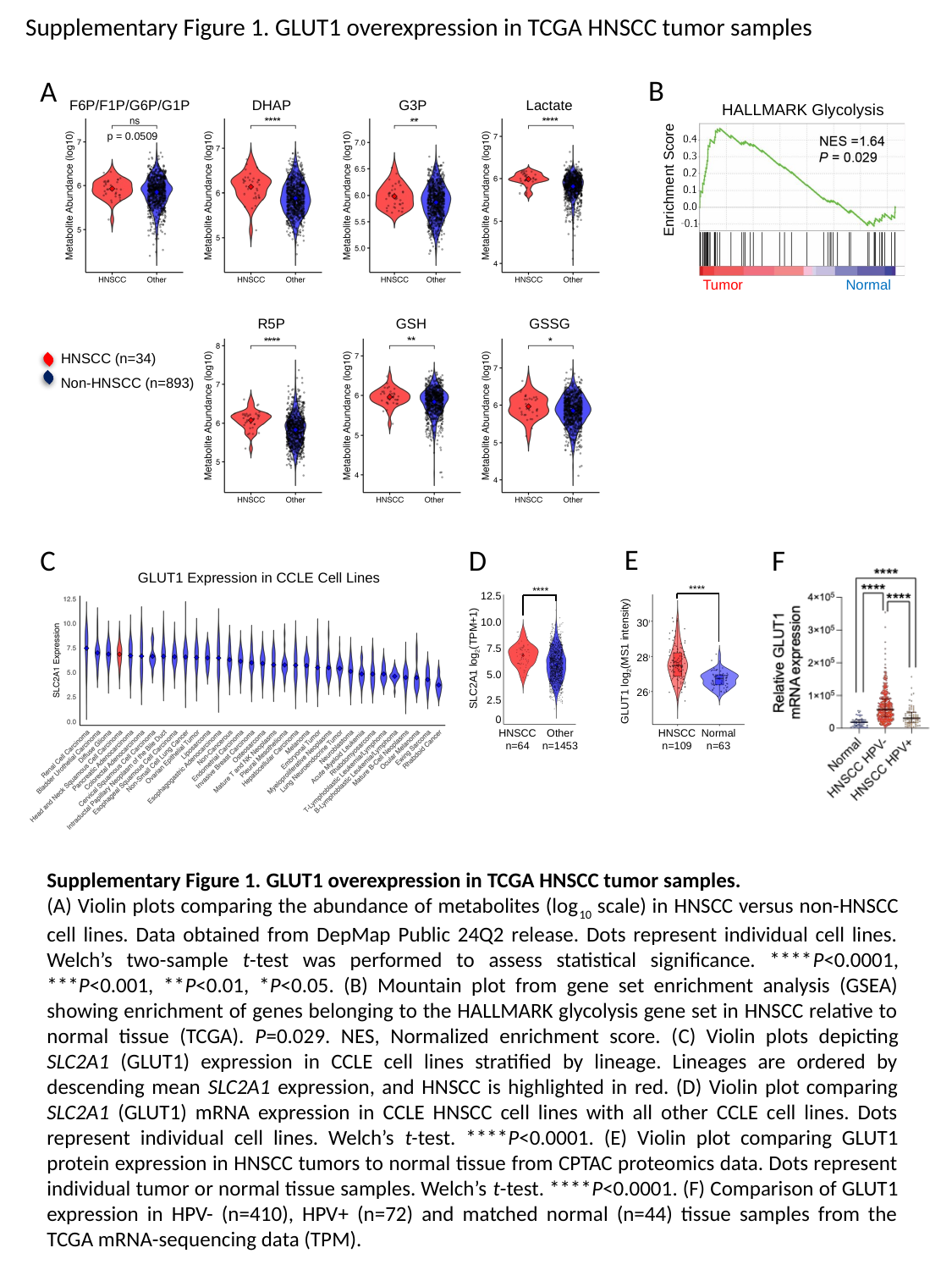

Supplementary Figure 1. GLUT1 overexpression in TCGA HNSCC tumor samples
B
A
F6P/F1P/G6P/G1P
DHAP
G3P
Lactate
HALLMARK Glycolysis
Enrichment Score
Tumor
Normal
****
****
**
p = 0.0509
R5P
GSH
GSSG
**
****
*
HNSCC (n=34)
Non-HNSCC (n=893)
E
C
D
F
GLUT1 Expression in CCLE Cell Lines
****
****
12.5
10.0
30
7.5
28
SLC2A1 log2(TPM+1)
GLUT1 log2(MS1 intensity)
5.0
26
2.5
0
HNSCC
n=64
Other
n=1453
HNSCC
n=109
Normal
n=63
Supplementary Figure 1. GLUT1 overexpression in TCGA HNSCC tumor samples.
(A) Violin plots comparing the abundance of metabolites (log10 scale) in HNSCC versus non-HNSCC cell lines. Data obtained from DepMap Public 24Q2 release. Dots represent individual cell lines. Welch’s two-sample t-test was performed to assess statistical significance. ****P<0.0001, ***P<0.001, **P<0.01, *P<0.05. (B) Mountain plot from gene set enrichment analysis (GSEA) showing enrichment of genes belonging to the HALLMARK glycolysis gene set in HNSCC relative to normal tissue (TCGA). P=0.029. NES, Normalized enrichment score. (C) Violin plots depicting SLC2A1 (GLUT1) expression in CCLE cell lines stratified by lineage. Lineages are ordered by descending mean SLC2A1 expression, and HNSCC is highlighted in red. (D) Violin plot comparing SLC2A1 (GLUT1) mRNA expression in CCLE HNSCC cell lines with all other CCLE cell lines. Dots represent individual cell lines. Welch’s t-test. ****P<0.0001. (E) Violin plot comparing GLUT1 protein expression in HNSCC tumors to normal tissue from CPTAC proteomics data. Dots represent individual tumor or normal tissue samples. Welch’s t-test. ****P<0.0001. (F) Comparison of GLUT1 expression in HPV- (n=410), HPV+ (n=72) and matched normal (n=44) tissue samples from the TCGA mRNA-sequencing data (TPM).
