# Supplementary material for: Targeting the Glucose–Insulin Link in Head and Neck Squamous Cell Carcinoma Induces Cytotoxic Oxidative Stress and Inhibits Cancer Growth
Source: Cancer Res Commun. 2025 Jun 6;5(6):921–38. doi: 10.1158/2767-9764.CRC-23-0506 (PMC12141995; doi:10.1158/2767-9764.CRC-23-0506)
Supplement: Figure S2 — Supplementary Figure 2 and legend [file crc-23-0506_figure_s2_suppsf2.pptx]

## Slide 1
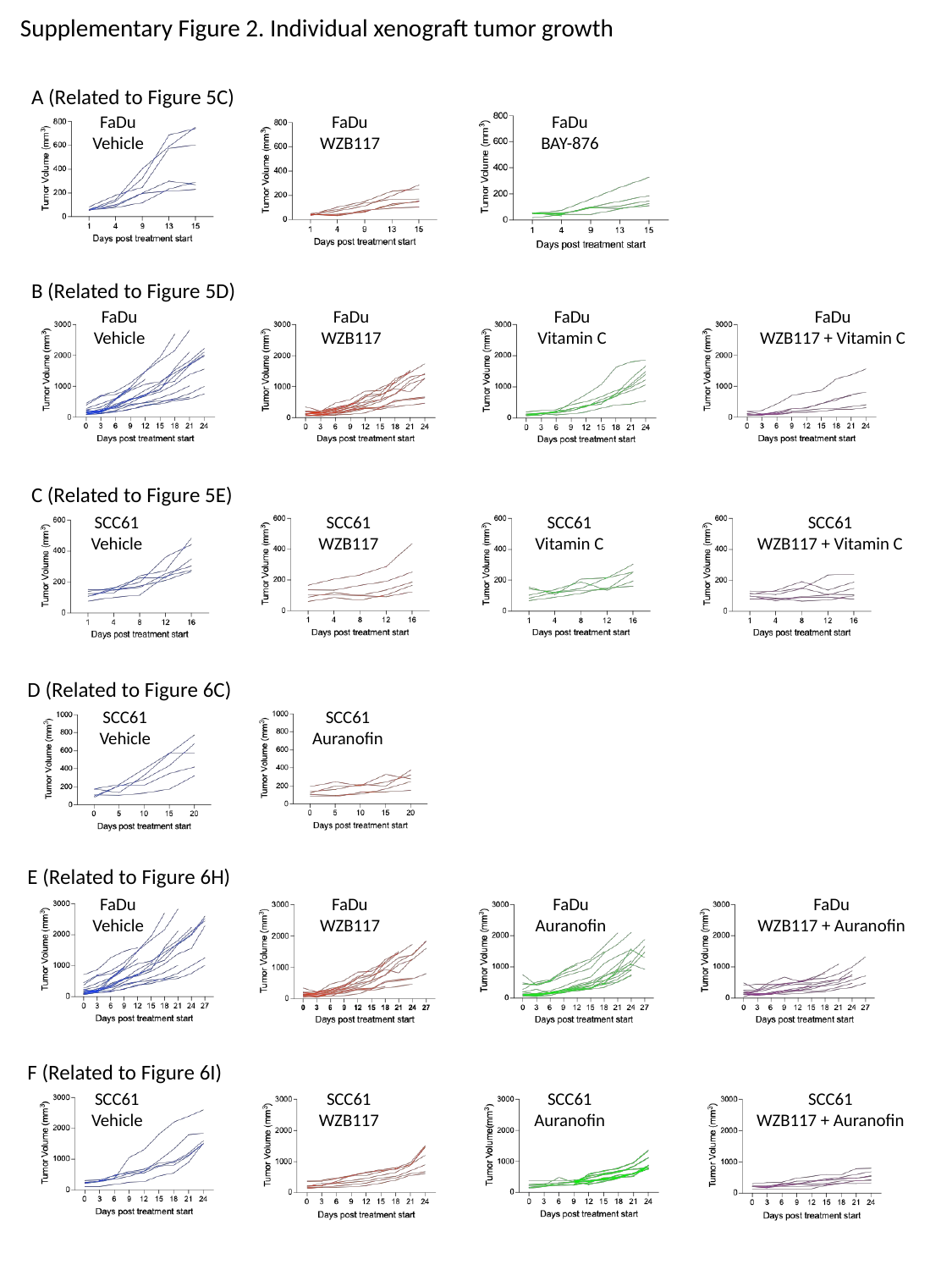

Supplementary Figure 2. Individual xenograft tumor growth
A (Related to Figure 5C)
FaDu
Vehicle
FaDu
WZB117
FaDu
BAY-876
B (Related to Figure 5D)
FaDu
Vehicle
FaDu
WZB117
FaDu
Vitamin C
FaDu
WZB117 + Vitamin C
C (Related to Figure 5E)
SCC61
Vehicle
SCC61
WZB117
SCC61
Vitamin C
SCC61
WZB117 + Vitamin C
D (Related to Figure 6C)
SCC61
Vehicle
SCC61
Auranofin
E (Related to Figure 6H)
FaDu
Vehicle
FaDu
WZB117
FaDu
Auranofin
FaDu
WZB117 + Auranofin
F (Related to Figure 6I)
SCC61
Vehicle
SCC61
WZB117
SCC61
Auranofin
SCC61
WZB117 + Auranofin

## Slide 2
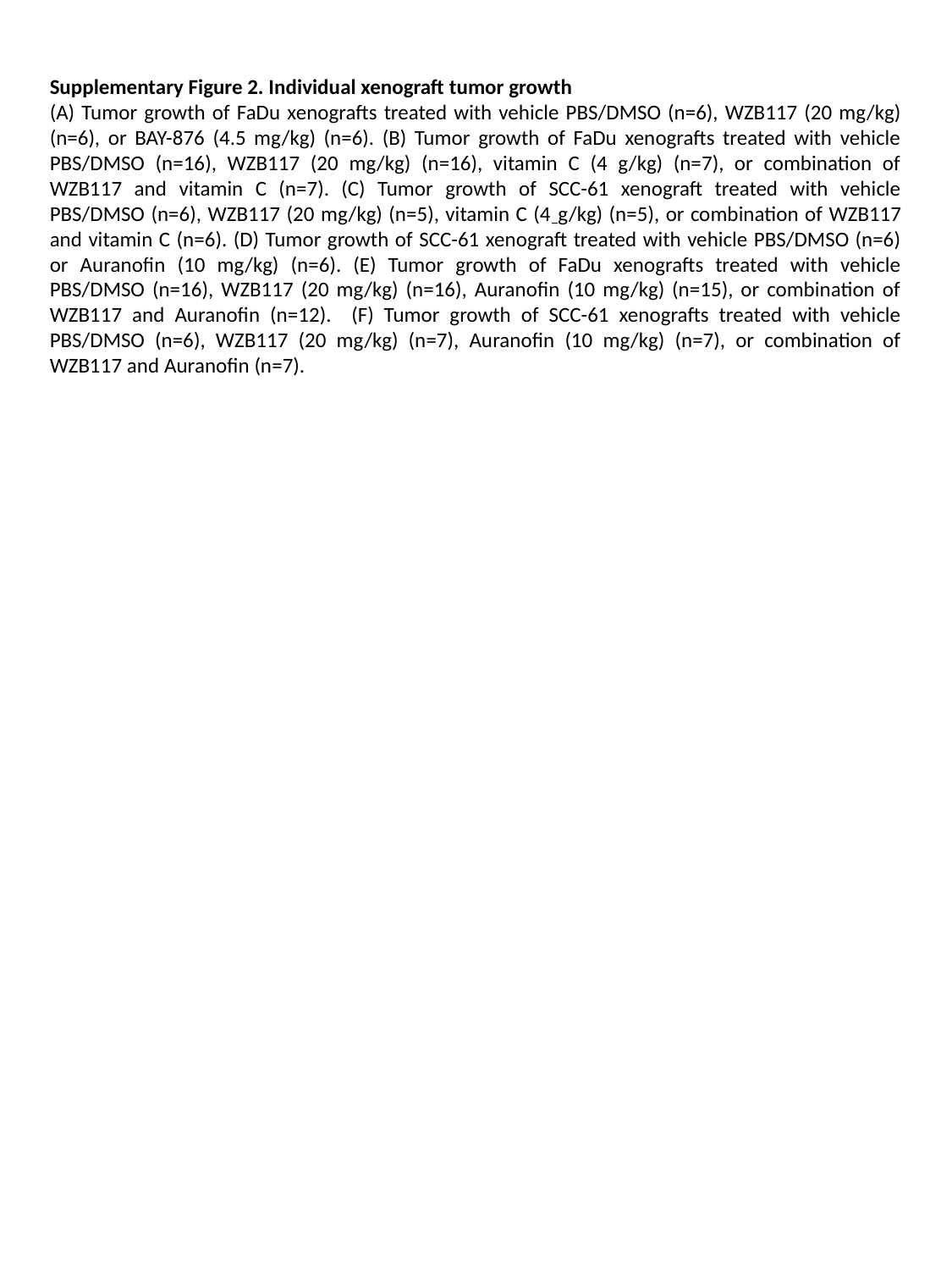

Supplementary Figure 2. Individual xenograft tumor growth
(A) Tumor growth of FaDu xenografts treated with vehicle PBS/DMSO (n=6), WZB117 (20 mg/kg) (n=6), or BAY-876 (4.5 mg/kg) (n=6). (B) Tumor growth of FaDu xenografts treated with vehicle PBS/DMSO (n=16), WZB117 (20 mg/kg) (n=16), vitamin C (4 g/kg) (n=7), or combination of WZB117 and vitamin C (n=7). (C) Tumor growth of SCC-61 xenograft treated with vehicle PBS/DMSO (n=6), WZB117 (20 mg/kg) (n=5), vitamin C (4 g/kg) (n=5), or combination of WZB117 and vitamin C (n=6). (D) Tumor growth of SCC-61 xenograft treated with vehicle PBS/DMSO (n=6) or Auranofin (10 mg/kg) (n=6). (E) Tumor growth of FaDu xenografts treated with vehicle PBS/DMSO (n=16), WZB117 (20 mg/kg) (n=16), Auranofin (10 mg/kg) (n=15), or combination of WZB117 and Auranofin (n=12). (F) Tumor growth of SCC-61 xenografts treated with vehicle PBS/DMSO (n=6), WZB117 (20 mg/kg) (n=7), Auranofin (10 mg/kg) (n=7), or combination of WZB117 and Auranofin (n=7).
