# Supplementary material for: Targeting the Glucose–Insulin Link in Head and Neck Squamous Cell Carcinoma Induces Cytotoxic Oxidative Stress and Inhibits Cancer Growth
Source: Cancer Res Commun. 2025 Jun 6;5(6):921–38. doi: 10.1158/2767-9764.CRC-23-0506 (PMC12141995; doi:10.1158/2767-9764.CRC-23-0506)
Supplement: Figure S3 — Supplementary Figure 3 and legend [file crc-23-0506_figure_s3_suppsf3.pptx]

## Slide 1
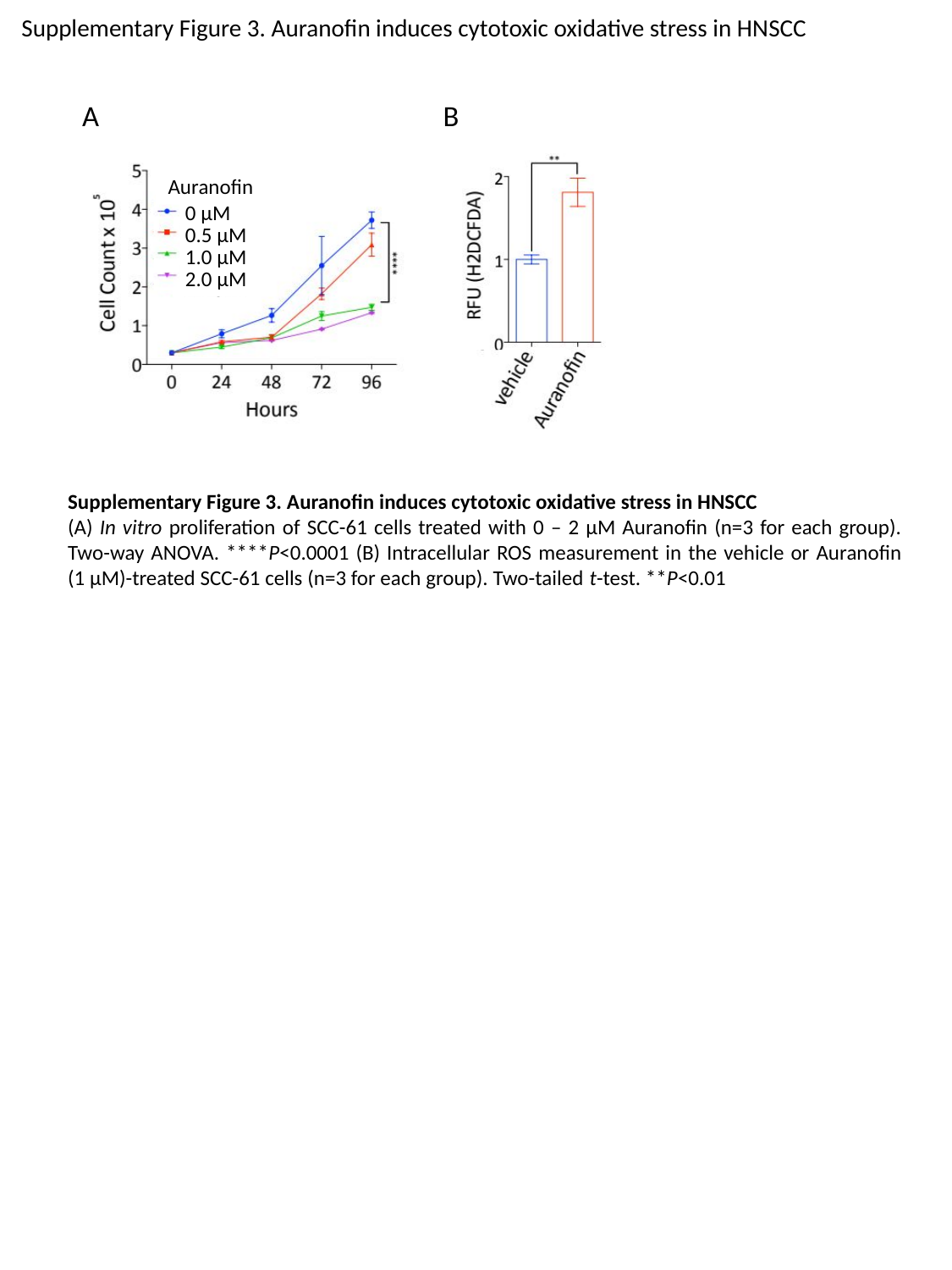

Supplementary Figure 3. Auranofin induces cytotoxic oxidative stress in HNSCC
A
B
Auranofin
0 μM
0.5 μM
1.0 μM
2.0 μM
Supplementary Figure 3. Auranofin induces cytotoxic oxidative stress in HNSCC
(A) In vitro proliferation of SCC-61 cells treated with 0 – 2 μM Auranofin (n=3 for each group). Two-way ANOVA. ****P<0.0001 (B) Intracellular ROS measurement in the vehicle or Auranofin (1 μM)-treated SCC-61 cells (n=3 for each group). Two-tailed t-test. **P<0.01
